# ID 249 - Relatórios de Análise Crítica para Apoiar a Tomada na Incorporação de Tecnologias na Saúde Suplementar

**DOI:** 10.5327/2237-9622.2025.v34s1.24

**Published:** 2025-11-25

**Authors:** Patrícia do Carmo Silva Parreira, Cecília Menezes Farinasso, Rafael Leite Pacheco, Ana Luiza Cabrera Martimbianco, Camila Monteiro Cruz, Carolina de Oliveira Cruz Latorraca, Isabela Porto de Toledo, Patrícia do Carmo Silva Parreira, Roberta Borges Silva, Verônica Colpani, Rachel Riera

**Keywords:** acessibilidade aos serviços de saúde, acesso a medicamentos essenciais e tecnologias em saúde, políticas e cooperação em ciência, tecnologia e inovação, saúde suplementar, ANS, ATS

## Abstract

**Introdução:**

Desde 2019, o Núcleo de Avaliação de Tecnologias em Saúde/Núcleo de Evidências do Hospital Sírio-Libanês (Nats/NEv-HSL) atua em colaboração com a Agência Nacional de Saúde Suplementar (ANS) na avaliação crítica de propostas de atualização (UAT) para o Rol de Procedimentos e Eventos em Saúde da Saúde Suplementar. O objetivo deste estudo foi apresentar as características dos relatórios de análises críticas (RAC) de propostas para atualização do Rol da ANS, elaborados de 2021 a 2024 pelo Nats/NEv-HSL, como parte de um projeto no âmbito do Programa de Apoio ao Desenvolvimento Institucional do Sistema Único de Saúde (Proadi-SUS).

**Método:**

Estudo descritivo conduzido pelo Nats/NEv-HSL e incluindo todos os RAC elaborados entre janeiro de 2021 agosto de 2024, identificados por meio de busca ativa em https://www.gov.br/ans/pt-br/acesso-a-informacao/participacao-da-sociedade/atualizacao-do-rol-de-procedimentos/propostas-de-atualizacao-do-rol-par.

**Resultados:**

Foram submetidas à ANS 106 UAT para incorporação no Rol, que seguiram para a realização de um RAC. Dos 106 RAC elaborados, 46 (43%) foram conduzidos pelo Nats/NEv-HS e se referiam a propostas de incorporação ao rol (em detrimento de propostas de alteração ou exclusão); 39 UAT (85%) foram referentes a medicamentos (em detrimento de procedimentos e testes diagnósticos); 31 UAT (67%) foram de condições oncológicas; e 28 (61%) resultaram em decisões favoráveis à incorporação da tecnologia.

**Conclusão:**

A maioria das propostas analisadas focaram medicamentos para tratamento de condições oncológicas e que houve maior frequência de decisões favoráveis. A construção de redes colaborativas entre as instituições brasileiras com expertise e/ou necessidade de processos pautados em ATS é fundamental para a sustentabilidade do sistema de saúde. A colaboração entre o Nats/NEv-HSL e a ANS tem contribuído para qualificar a tomada de decisão na saúde suplementar e garantir um alinhamento metodológico e processual no cenário da incorporação de tecnologias no Brasil, considerando o sistema público e a saúde suplementar.

